# ISKpn28 transposition and genomic alterations in λ-Red recombinants of hypervirulent *Klebsiella pneumoniae*: Whole-genome sequencing and phenotypic analysis

**DOI:** 10.1080/21505594.2026.2732382

**Published:** 2026-11-01

**Authors:** Seong Hun Jeong, Ju Yeong Lee, Keon Young Lee, Miri Hyun, Ji Yeon Lee, Michael McClelland, Jin Kyung Kim, Hyun ah Kim

**Affiliations:** a Department of Infectious Diseases, Keimyung University Dongsan Hospital, Keimyung University School of Medicine, Keimyung University, Daegu, Republic of Korea; b Department of Microbiology, Keimyung University School of Medicine, Daegu, Republic of Korea; c Department of Microbiology and Molecular Genetics, University of California, Irvine, CA, USA

**Keywords:** Hypervirulent *Klebsiella pneumoniae*, λ-Red recombination, phenotypic variation, whole-genome sequencing, ISKpn28, secondary genomic alteration

## Abstract

The λ-Red recombination system is commonly used to investigate gene function in *Klebsiella pneumoniae*; however, unintended secondary genomic alterations in recombinant strains may remain undetected by locus-specific validation methods such as polymerase chain reaction and Sanger sequencing. Following *rmpA* mutant construction in the clinical hypervirulent *K. pneumoniae* (hvKp) strain K255, two recombinant derivatives showed identical results on locus-specific validation, confirming the intended *rmpA* replacement. However, the strains differed in uronic acid content and interaction with murine macrophages. We therefore performed whole-genome sequencing (WGS) incorporating both short- and long-read data to investigate potential secondary genomic alterations. Both recombinant derivatives harbored an additional chromosomal copy of ISKpn28 within the *nlpD* gene. The K255Δ*rmpA*-1 strain additionally exhibited a chromosomal deletion of 55,652 base pairs, whereas K255Δ*rmpA*-2 harbored an ISKpn74-associated plasmid inversion. These findings provide genomic evidence of ISKpn28 transposition in recombinant derivatives of the clinical hvKp strain K255, and highlight the potential value of WGS incorporating long-read sequencing for assessing genome-wide integrity in λ-Red-derived recombinant hvKp strains harboring ISKpn28.

## Introduction

Hypervirulent *Klebsiella pneumoniae* (hvKp) was initially reported predominantly in East Asia and has emerged as a major community-acquired pathogen. In recent years, hvKp has also been recognized across other continents, including North America, Europe, and Africa, underscoring its global clinical relevance. Recent meta-analyses have reported a substantial global prevalence of hvKp among clinical *K. pneumoniae* isolates, with pooled prevalence estimates ranging from approximately 4.9% to 27.2% across regions [1,2].

Unlike classical *K. pneumoniae* strains, hvKp can cause invasive infections even in healthy individuals and is associated with severe diseases, including liver abscess, meningitis, pneumonia, endophthalmitis, and sepsis, often accompanied by metastatic infections. A hallmark of hvKp is pronounced mucoviscosity, which facilitates hematogenous dissemination and contributes to clinical severity. The *rmpA* gene, a key regulator of mucoviscosity, is closely associated with the mucoid phenotype characteristic of hvKp and is thought to enhance virulence [3,4]. To investigate the role of *rmpA* in the clinical hvKp strain K255, we generated recombinant derivatives in which the plasmid-borne *rmpA* gene was replaced with a tetracycline-resistance cassette using the λ-Red recombination system [5].

Among the resulting colonies grown under the same tetracycline-selection conditions, we observed two distinct colony types. Despite carrying the same *rmpA* replacement with a tetracycline-resistance cassette, the two recombinant strains exhibited markedly different phenotypes and interactions with murine macrophages. These unexpected differences prompted whole-genome sequencing (WGS) to investigate potential secondary genomic alterations. WGS revealed genomic alterations in the two recombinant derivatives relative to the parental K255 strain, including an additional insertion sequence (IS) copy, a large chromosomal deletion, and plasmid inversion.

In this study, we aimed to compare the in vitro phenotypic characteristics of two λ-Red-derived recombinants of the hvKp strain K255 and to characterize the unexpected genomic alterations identified in these derivatives using WGS incorporating both short- and long-read data.

## Materials and methods

This study did not involve human participants or animals requiring ethical approval.

### Bacterial strains and culture conditions

The hvKp strain, K255, used in this study was a clinical bloodstream isolate recovered from a patient with a pyogenic liver abscess at Keimyung University Dongsan Hospital, Daegu, Republic of Korea. Kleborate [6,7] analysis identified K255 as capsule locus KL2 and sequence type ST65.

Unless otherwise specified, the wild-type (WT) strain, K255, was cultured in standard Luria–Bertani (LB) broth and agar (BD, NJ, USA), whereas recombinant strains were cultured in LB broth and agar supplemented with tetracycline (15 μg/mL). Bacterial cultures were incubated at 37°C on agar plates or in liquid cultures shaken at 200 rpm.

### Recombinant strain construction

Detailed information on the bacterial strains, plasmids, and primers used in this study is provided in Tables S1–S3. K255 cells were initially transformed with the temperature-sensitive plasmid pKD46, which carries a spectinomycin resistance gene by electroporation. Competent cells were prepared by inoculating a glycerol stock of pKD46-transformed K255 into 23 mL LB medium supplemented with spectinomycin (100 μg/mL) and incubating at 30°C with shaking at 200 rpm for 1 h. After incubation, L-arabinose was added to a final concentration of 0.02% to induce λ-Red recombinase expression [8]. Cells were incubated again with shaking at 30°C until reaching an optical density of approximately 0.48 at 600 nm (OD_600_).

Cells were chilled on ice for 30 min, then centrifuged at 6,000 rpm for 5 min at 4°C. The supernatant was carefully removed, and the pellet was washed gently twice using 20 mL of ice-cold 10% glycerol. Subsequently, the bacterial pellet was resuspended in 200 μL of ice-cold 10% glycerol. Cells were aliquoted in 65-μL volumes, immediately frozen on dry ice, and stored at −80°C until further use.

To replace plasmid-borne *rmpA* with a tetracycline-resistance cassette, approximately 1.5 µg of purified polymerase chain reaction (PCR) product containing the tetracycline resistance gene cassette flanked by homologous sequences targeting the *rmpA* gene was added to a 65-µL aliquot of freshly thawed electrocompetent cells on ice. After incubation on ice for 30 min, the mixture was transferred into a pre-chilled 0.1-cm electroporation cuvette (Bio-Rad, CA, USA). Electroporation was conducted with a Bio-Rad Micropulser (Bio-Rad, CA, USA) at 1.8 kV, achieving a pulse duration of approximately 6.0 ms. Immediately following electroporation, cells were transferred to 950 µL of super optimal broth with catabolite repression (SOC) medium [9] and allowed to recover at 37°C with shaking at 200 rpm for 50 min. Cells were then plated onto LB agar plates containing tetracycline (15 μg/mL) for selection and incubated overnight at 37°C. Colonies were screened by colony PCR to confirm replacement of *rmpA*, using KAPA2G Robust HotStart ReadyMix (Roche, Basel, Switzerland) according to the manufacturer’s instructions.

### Genomic DNA extraction and WGS

Genomic DNA was independently extracted from each strain for downstream analyses. Bacteria were grown in 5 mL LB broth in test tubes using a shaking incubator. DNA extraction was performed using the GeneAll® GenEx™ Cell Sx kit (GeneAll, Seoul, Republic of Korea) according to the manufacturer’s protocol. Short- and long-read data were generated by Macrogen Inc. (Seoul, Republic of Korea) using the Illumina NovaSeq X platform with a TruSeq Nano DNA (350) library and the PacBio Revio platform with a PacBio Microbial Library, respectively.

### Validation PCR and agarose gel electrophoresis

PCR amplification was performed in 0.2-mL tubes using a Biometra TAdvanced thermal cycler (Analytik Jena, Jena, Germany). DNA polymerase, reaction buffers, MgCl_2_ (Cat. No. M8295), and dNTPs (Part No. U151B) were obtained from Promega (Madison, WI, USA) and used according to the manufacturer’s instructions. PCR products were separated on 1.5% agarose gels prepared in 1× TAE buffer and stained with EcoDye™ (Biofact, Daejeon, Republic of Korea). Electrophoresis was conducted using a Mupid-2Plus system (Advance, Tokyo, Japan) at 100 V for 40 min. DNA ladders (1 kb, Cat. No. D-1040; 100 bp, Cat. No. D-1030; Bioneer, Daejeon, Republic of Korea) were used for fragment-size estimation, and gels were visualized under UV illumination using an SH-523 imaging system (SHST, China). Detailed PCR conditions are described in Table S3.

### Validation of *rmpA* replacement by PCR and Sanger sequencing

Genomic DNA from K255, K255Δ*rmpA*-1, and K255Δ*rmpA*-2 was used as the template. For size-diagnostic PCR, a primer pair flanking the targeted *rmpA* region was used, with expected amplicon sizes of 936 base pairs (bp) for the parental K255 strain and 2,575 bp for the recombinant derivatives. The size-diagnostic PCR products obtained from K255Δ*rmpA*-1 and K255Δ*rmpA*-2 were subjected to Sanger sequencing to verify the upstream and downstream junctions between the flanking plasmid sequences and the tetracycline-resistance cassette in each recombinant derivative.

The upstream recombination junction was further examined using a junction-specific primer pair, which generated an expected 220-bp amplicon in the recombinant derivatives. Table S3 provides details on primer sequences, primer combinations, and detailed PCR conditions.

### String test

The string test was performed using colonies grown at 37°C on antibiotic-free LB agar or LB agar supplemented with tetracycline (15 μg/mL). Three spatially separated colonies from the same plate were individually touched with a sterile 1-mm inoculating loop and lifted vertically. The maximum length of the viscous string formed before breaking was measured for each colony.

### Mucoviscosity assay

Mucoviscosity was evaluated using a sedimentation resistance assay, as previously described [4]. Overnight cultures were grown in LB broth without or with tetracycline (15 μg/mL). Before measurement, cultures were thoroughly vortexed and repeatedly pipetted to obtain uniform suspensions, and no obvious aggregation or flocculation was observed. The OD_600_ of each culture was measured after a 1:10 dilution in phosphate-buffered saline (PBS, Welgene, Gyeongsangbuk-do, Republic of Korea) using a spectrophotometer (WPA S2000, Biochrom, UK) with a 1-cm path-length cuvette. Based on these measurements, the cultures were adjusted to an OD_600_ of 1.0 with PBS in sterile 2-mL microcentrifuge tubes and centrifuged at 1,000 × g for 5 min. Subsequently, 900 μL of the supernatant was transferred to a new cuvette, and its OD_600_ was recorded as an index of sedimentation resistance.

### Uronic acid assay

Extracellular polysaccharide content was quantified using a uronic acid colorimetric assay as previously described [4], with minor modifications. Although the original protocol recommended cultivation in low-salt LB medium, strains in this study were grown in normal LB medium. Overnight cultures were grown in LB broth without or with tetracycline (15 μg/mL). Aliquots of the overnight cultures were adjusted to an OD_600_ of 1.0, serially diluted in PBS, and plated on LB agar to determine colony-forming unit (CFU) counts. Viable counts of the original overnight cultures were back-calculated by accounting for both the dilution used to adjust the cultures to an OD_600_ of 1.0 and the subsequent serial dilution factors. All centrifugation steps during polysaccharide purification were performed at 16,000 × *g*. Uronic acid standards were prepared using D-glucuronic acid (Sigma, St Louis, USA). Extracellular polysaccharides were extracted by treatment with 1% Zwittergent 3–14 (Millipore, MA, USA) in 100 mM citric acid (Sigma-Aldrich, St Louis, USA) buffer adjusted to pH 2 with 1 N hydrochloric acid and subsequently precipitated with absolute ethanol. The resulting polysaccharide pellets were rehydrated in distilled water, and uronic acid content was quantified using a colorimetric assay with 0.0125 M sodium tetraborate (Sigma-Aldrich, St Louis, USA) in concentrated sulfuric acid, and 0.3% 3-phenylphenol (Sigma-Aldrich, St Louis, USA) in 0.125 M NaOH. Absorbance at 520 nm was measured using a spectrophotometer with a 1-cm path-length cuvette. Uronic acid concentrations were normalized to the corresponding CFU values.

### Phagocytosis and adhesion assays

Unless otherwise specified, RAW264.7 (TIB-71, ATCC, USA) cells were maintained in DMEM (Welgene, Gyeongsangbuk-do, Republic of Korea) supplemented with 5% fetal bovine serum (Welgene, Gyeongsangbuk-do, Republic of Korea) at 37°C in a humidified atmosphere containing 5% CO_2_. RAW264.7 cells were seeded in 12-well plates at a density of 7 × 10^5^ cells/well and infected with K255Δ*rmpA*-1 or K255Δ*rmpA*-2 at a multiplicity of infection of 40 and incubated for 1 h.

For phagocytosis assays, following infection, cells were washed with PBS supplemented with 100 μg/mL gentamicin (Gibco, NY, USA) to remove extracellular bacteria and incubated for an additional 30 min in fresh gentamicin-containing culture medium. Following incubation, cells were washed again with gentamicin-containing PBS, lysed with 100 μL of 2% saponin (Sigma-Aldrich, St Louis, USA) prepared in distilled water (DW), followed by addition of 100 μL DW and 15 min incubation. Lysates were diluted in PBS, plated on LB agar, incubated overnight, and colony-forming units (CFU) were enumerated.

For adhesion assays, cells were washed three times with PBS to eliminate non-adherent bacteria. Host cells were then disrupted in 200 μL of 0.25% Triton X-100 (Thermo Fisher Scientific, MA, USA) for 15 min, and released bacteria were recovered, serially diluted, and quantified by plating on LB agar.

### Bacterial growth curve

Bacterial strains were inoculated into 2 mL of LB broth in sterile 17 × 100 mm culture tubes and incubated with shaking for 18 h. The overnight cultures were adjusted to an OD_600_ of 0.05 using fresh LB broth, and 2 mL of each suspension was further incubated with shaking. At each time point (2, 4, 6, 8, 10, and 12 h), 100 µL aliquots were collected for OD_600_ measurement using uncoated 96-well plates and a Synergy HTX multi-mode microplate reader (BioTek, VT, USA).

### Genome assembly and annotation

All analyses were conducted on a local workstation running Ubuntu 20.04.6 LTS, with tools managed with *Conda* (v24.11.3). Quality assessment of the raw reads was conducted using *FastQC* (v0.12.1) [10]. Raw read preprocessing for the strains was conducted using *fastp* (v0.23.4) for short reads [11] and *Filtlong* (v0.2.1) [12] for long reads. Hybrid genome assemblies were generated using *Unicycler* (v0.5.1), following the workflow recommended by Wick et al. [13,14]. Annotation of the completed circular genome and plasmid was conducted using NCBI PGAP v2024-07–18.build7555 [15] within a Docker Desktop v4.37.1 [16] environment. For all three strains, the organism name was set as “*Klebsiella pneumoniae*.” Additionally, for the recombinants, the “*–ignore-all-errors*” option was specified. As a final quality-control step, the completed genome assemblies were screened for homopolymeric tracts containing more than 11 consecutive identical nucleotides (A, T, G, or C).

### Removal of an anomalous read before final genome assembly of K255Δ*rmpA*-2

During preliminary hybrid genome assembly of K255Δ*rmpA*-2, an anomalous sequence was incorporated into the assembled genome. The corresponding read contained a homopolymeric tract of 50 consecutive cytosine nucleotides and lacked support from any other sequencing reads. It was therefore classified as an anomalous read. General low-complexity filtering was initially evaluated; however, filtering stringent enough to remove this read resulted in substantial loss of sequence data. To minimize unnecessary data loss, only this single anomalous read was excluded before the final genome assembly. The corresponding read was manually removed using the Linux command “*awk*” for text-based filtering. The cleaned FASTQ files were then reconstructed into paired-end format using *BBMap* (v39.15) [17] and subsequently preprocessed using the same approach described above. The two unpaired read files generated from the *BBMap* and *fastp* steps were then combined into a single file using the Linux “*cat”* command for file concatenation. The resulting files were then used for hybrid genome assembly using *Unicycler*. Information on the excluded read is provided in Table S4. The table also presents assembly metrics before and after exclusion of the anomalous read. The assemblies generated before and after exclusion of the anomalous read have been deposited in Figshare [18].

### Genome analysis

The resulting GFF files from PGAP were converted to spreadsheet format and processed in *R* (v4.4.1) [19] using the *tidyverse* package [20] to sort and inspect gene content differences across the three strains.

For analysis of ISKpn28 loci, flanking sequences were examined to identify direct repeats (DRs) surrounding each locus. Based on the characteristic 5-bp DR generated by ISKpn28 insertion [21], BLAST [22] searches were conducted against the NCBI database, with the search set restricted to “*Klebsiella pneumoniae* (taxid: 573)” using 1,000-bp flanking sequences from which one copy of the DR was excluded. Only BLAST hits showing ≥ 99% identity and 100% query coverage were retained for downstream analyses. Basic genome sequence inspection, comparison, alignment, and visualization were performed using *Benchling* [23].

### Validation of genomic alterations by PCR and Sanger sequencing

The ISKpn28-associated junctions identified by WGS were validated by PCR amplification and Sanger sequencing. Genomic DNA previously extracted for WGS was used as the template for junction-specific PCR. In K255Δ*rmpA*-1, the upstream *nlpD*-ISKpn28 junction and the downstream ISKpn28-*hypD* deletion junction were examined. In K255Δ*rmpA*-2, the upstream and downstream *nlpD*-ISKpn28 junctions were examined. PCR results obtained from K255 were included as a WT control.

To validate the ISKpn74-associated plasmid inversion in K255Δ*rmpA*-2, configuration-specific junction PCR was performed. Separate primer combinations were used to distinguish the parental and inverted plasmid configurations across the ISKpn74 copies flanking the inverted region in K255, K255Δ*rmpA*-1, and K255Δ*rmpA*-2.

To assess the clonal fixation of these genomic alterations, 10 colonies from each of K255Δ*rmpA*-1 and K255Δ*rmpA*-2 were examined. DNA templates from individual colonies were prepared as previously described [24].

Representative PCR products from the recombinant strains were sequenced by Macrogen Inc. (Seoul, Republic of Korea). Table S3 provides primer sequences and detailed PCR conditions.

### Assessment of the predicted plasmid inversion by long-read mapping and read-level analysis

To further examine the ISKpn74-associated plasmid inversion, raw PacBio HiFi reads from K255, K255Δ*rmpA*-1, and K255Δ*rmpA*-2 were mapped to the K255 genome assembly as the reference using minimap2 (v2.28-r1209) [25] with the *map-hifi* preset. Mapping, BAM conversion, and sorting were performed using the following command:

minimap2 -t 8 -ax map-hifi K255.fa <file_prefix>_HiFi.fastq.gz | \

samtools view -bS - | \

samtools sort -@ 8 -f -<file_prefix>.bam.

Here, <file_prefix> represents the filename prefix used for each sequencing dataset. The resulting BAM files were indexed using SAMtools (v0.1.19-96b5f2294a) [26]. Read alignments were visually inspected using Integrative Genomics Viewer (IGV, v2.19.4) [27].

Alignment records containing the SA:Z tag were identified and counted within four 5-kb regions encompassing the outer and inner flanks of the two inversion boundaries using SAMtools. The numbers of SA-tagged alignments were compared among the three strains to assess read-level support for the predicted plasmid inversion. The K255 reference coordinates examined in all three strains are provided in Table S5.

### Statistical analysis

Statistical analyses were performed using GraphPad Prism, version 8.4.0 (San Diego, CA, USA). Differences between the two recombinant strains were analyzed using two-tailed unpaired t-tests. For growth-curve analysis, the area under the curve (AUC) was calculated for each biological replicate, and AUC values were compared between the two recombinant strains using a two-tailed unpaired t-test. Unless otherwise specified, all experiments were performed in three independent biological replicates, and data are expressed as mean ± SD (ns, not significant; *p* ≤ 0.05 [*], *p* ≤ 0.01 [**], *p* ≤ 0.001 [***], *p* ≤ 0.0001 [****]).

## Results

### Validation of *rmpA* replacement in recombinant strains

Following *rmpA* replacement, two phenotypically distinct colony types were observed, and representative colonies were designated K255Δ*rmpA*-1 and K255Δ*rmpA*-2.

Size-diagnostic PCR yielded the expected 936-bp amplicon in parental K255 and 2,575-bp amplicons in K255Δ*rmpA*-1 and K255Δ*rmpA*-2. Junction-specific PCR further verified the intended *rmpA* replacement in both recombinant derivatives (Figure 1). Sanger sequencing of the 2,575-bp PCR products confirmed the predicted upstream and downstream recombination junction sequences in both recombinant derivatives. The corresponding raw Sanger sequencing files and recombination-junction sequence alignments are available in the Figshare repository [18].

**Figure 1.**
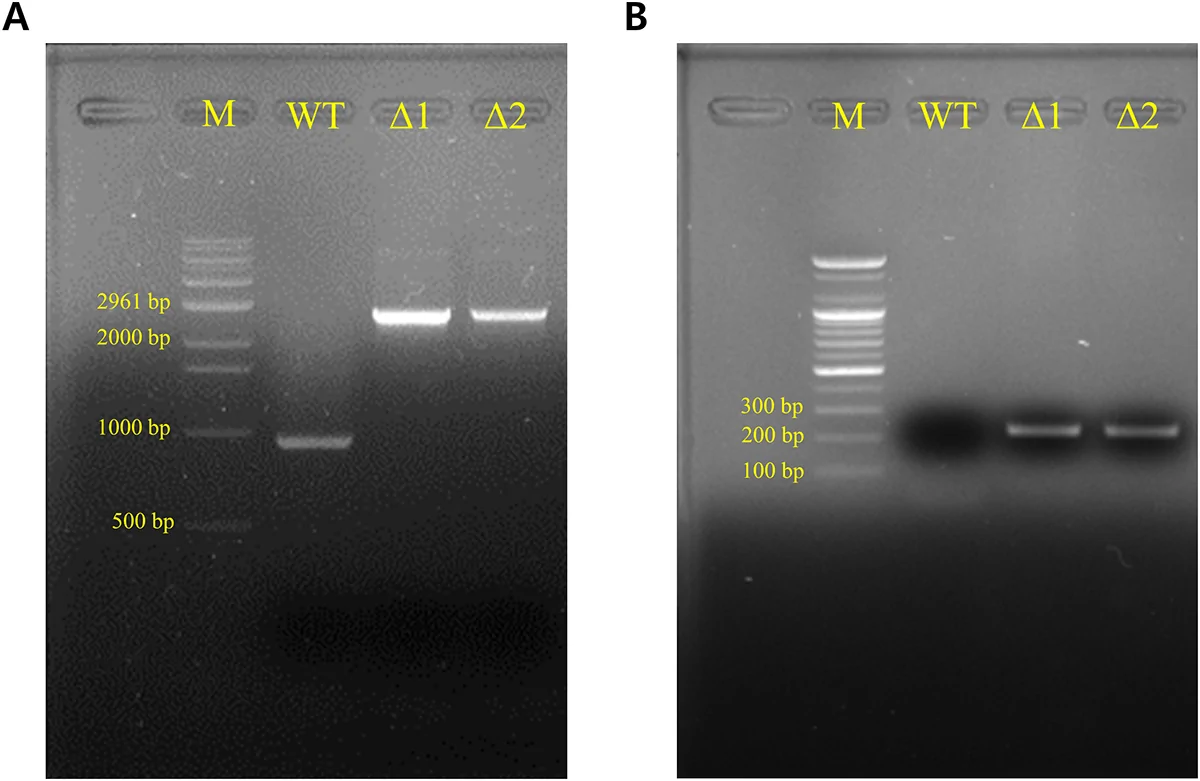
PCR validation of *rmpA* replacement in recombinant strains. (A) Size-diagnostic PCR using primers flanking the targeted *rmpA* region produced the expected 936-bp amplicon in parental K255 (WT) and 2,575-bp amplicons in K255Δ*rmpA*-1 (Δ1) and K255Δ*rmpA*-2 (Δ2). (B) Junction-specific PCR targeting the upstream recombination junction produced the expected 220-bp amplicon in Δ1 and Δ2 but not in WT. M, DNA size marker.

### Distinct phenotypic characteristics of recombinant strains

The two *rmpA*-replaced recombinant derivatives exhibited phenotypic differences in mucoviscosity and uronic acid content. Under tetracycline-free conditions, no significant difference in mucoviscosity was observed between the two derivatives, whereas K255Δ*rmpA*-2 exhibited higher mucoviscosity than K255Δ*rmpA*-1 under tetracycline-containing conditions (Figure 2(A,B)). Consistent with the mucoviscosity assay, the string test showed no appreciable string formation by either derivative under tetracycline-free conditions, whereas K255Δ*rmpA*-2 produced longer strings than K255Δ*rmpA*-1 under tetracycline-containing conditions (Figure S1).

**Figure 2.**
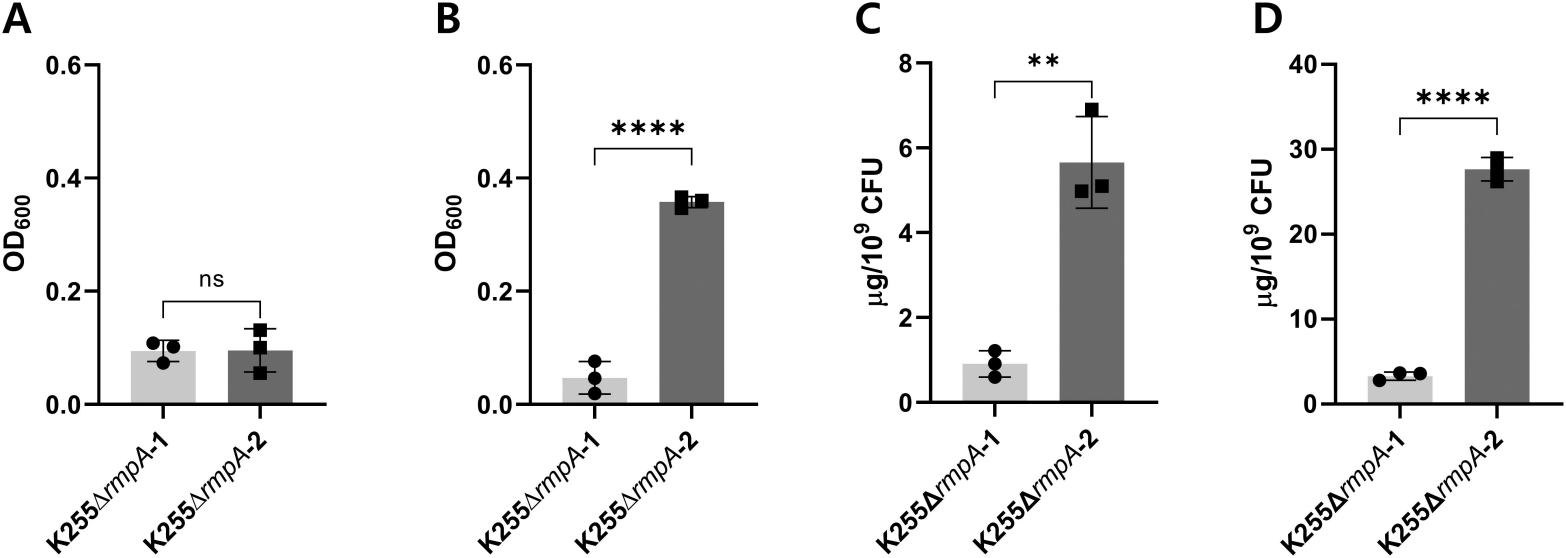
Phenotypic comparison of *rmpA*-replaced recombinant strains under tetracycline-free and tetracycline-containing conditions. (A, B) Mucoviscosity assessed by sedimentation resistance under tetracycline-free (A) and tetracycline-containing conditions (15 μg/mL) (B). (C, D) Uronic acid content normalized to CFU under tetracycline-free (C) and tetracycline-containing conditions (15 μg/mL) (D). Data are presented as mean ± SD from three independent biological replicates.

In contrast, uronic acid content was higher in K255Δ*rmpA*-2 under both tetracycline-free and tetracycline-containing conditions (Figure 2(C,D)).

### Differences in phagocytosis and adhesion between recombinant strains

Phagocytosis and adhesion to murine macrophages were assessed as virulence-associated traits using recombinant strains grown under tetracycline-containing conditions (Figure 3(A,B)). In the phagocytosis assay, K255Δ*rmpA*-2 yielded significantly lower CFU counts following phagocytic uptake than K255Δ*rmpA*-1. Adhesion assays revealed markedly higher adhesion by K255Δ*rmpA*-1 compared with K255Δ*rmpA*-2, indicating distinct macrophage interactions.

**Figure 3.**
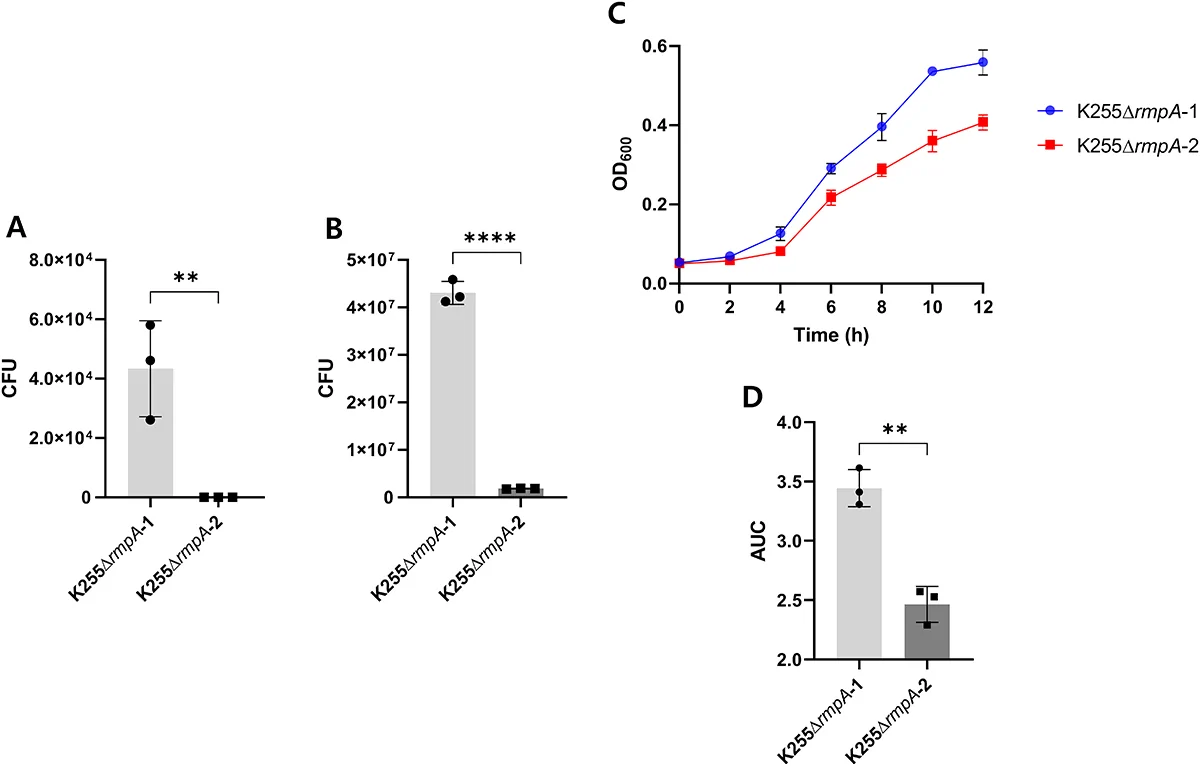
Virulence-associated phenotypes and growth characteristics of recombinant strains. (A) Phagocytosis and (B) adhesion assays in RAW 264.7 cells. (C) Growth curves of K255Δ*rmpA*-1 and K255Δ*rmpA*-2 strains. (D) Area-under-the-curve (AUC) analysis of the growth curves. Data are presented as mean ± SD from three independent biological replicates. Recombinant strains were grown under tetracycline-containing conditions.

### Growth comparison of recombinant strains

Growth curves of the recombinant strains under tetracycline-containing conditions are shown in Figure 3(C). K255Δ*rmpA*-1 exhibited a higher growth-curve AUC than K255Δ*rmpA*-2 (Figure 3(D)).

### WGS and structural analysis of recombinant strains

WGS and annotation were performed for all three strains; results are summarized in Table 1. During genome annotation, ISKpn28 sequences lacking a canonical stop codon were automatically labeled as “IS481 family transposase” and “pseudo.” These loci were manually verified using BLASTn searches against the ISfinder database.

**Table 1.** Summary of genome assembly and annotation results.

	K255	K255Δ*rmpA*-1	K255Δ*rmpA*-2
Chromosome length (bp)	5,252,471	5,198,010	5,253,470
Plasmid length (bp)	234,954	236,601	236,593
Coding genes	5,099	5,042 (+3)*	5,099 (+1)*
Pseudo genes	141	147	143
RNA genes	123	123	123
Total genes	5,363	5,312 (+3)*	5,365 (+1)*
ISKpn28 loci (Chromosome/Plasmid)	9/1	10/1	10/1

*“+” indicates loci where hypothetical proteins were not annotated by PGAP but identical DNA sequences were identified at corresponding genomic positions (see Table S6).

The recombinant strain K255Δ*rmpA*-1 exhibited a large chromosomal deletion spanning coordinates 1,066,885–1,122,537 of the WT genome, corresponding to a loss of 55,652 bp (Figure 4). Annotated gene differences are summarized in Table 2. This deletion resulted in the complete loss of 50 annotated genes, with five additional genes identified as pseudogenes relative to the WT.

**Figure 4.**
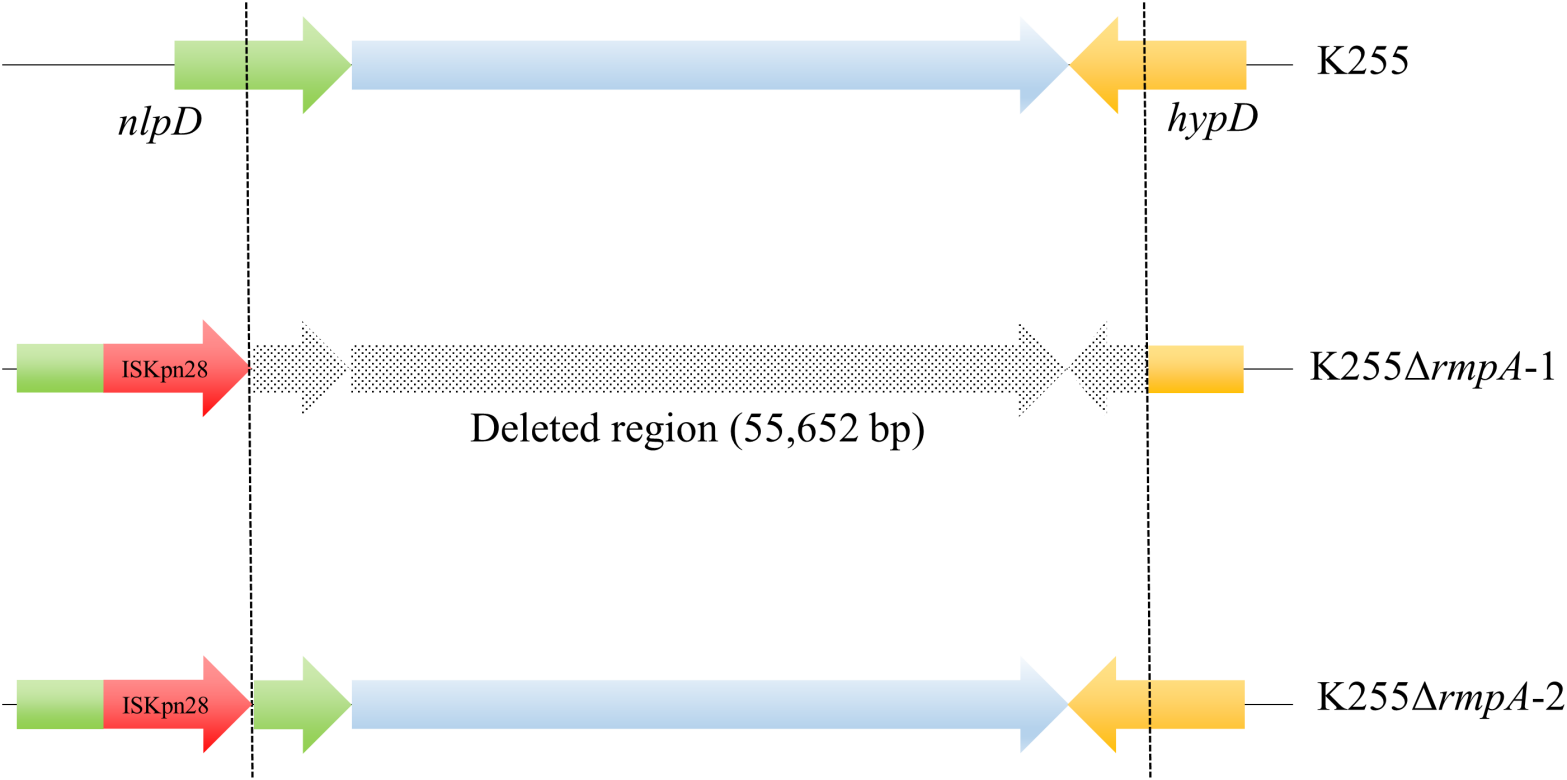
Schematic representation of genomic alterations in the recombinant strains. In parental K255, the region between *nlpD* and *hypD* is intact. In K255Δ*rmpA*-1, ISKpn28 is located within *nlpD*, with a 55,652-bp chromosomal deletion spanning *nlpD* to *hypD*. K255Δ*rmpA*-2 also harbors ISKpn28 within *nlpD* but retains the remainder of this region. Arrows indicate genes and their orientations, the red arrow indicates ISKpn28, and the dotted region indicates the deleted segment.

**Table 2.** Annotation difference comparison.

Product	K255		K255Δ*rmpA*-1		K255Δ*rmpA*-2	
	Start	Stop	Start	Stop	Start	Stop
**Chromosome**						
gallate dioxygenase	56,371	57,633	56,371*^2)^	57,634*	56,371	57,633
LysM peptidoglycan-binding domain-containing protein	–^1)^	–	1,066,297*	1,066,887*	1,066,296*	1,066,886*
IS481 family transposase (ISKpn28)	–	–	1,066,944*	1,067,978*	1,066,943	1,068,016
murein hydrolase activator NlpD	1,066,296	1,067,432	–	–	1,067,982*	1,068,533*
RNA polymerase sigma factor RpoS	1,067,590	1,068,582	–	–	1,068,691	1,069,683
DUF4440 domain-containing protein	1,068,635	1,069,012	–	–	1,069,736	1,070,113
MFS transporter	1,069,009	1,070,211	–	–	1,070,110	1,071,312
LysR substrate-binding domain-containing protein	1,070,320	1,071,207	–	–	1,071,421	1,072,308
MarR family winged helix-turn-helix transcriptional regulator	1,071,216	1,071,623	–	–	1,072,317	1,072,724
non-oxidative hydroxyarylic acid decarboxylases subunit B	1,071,797	1,072,390	–	–	1,072,898	1,073,491
non-oxidative hydroxyarylic acid decarboxylases subunit C	1,072,390	1,073,817	–	–	1,073,491	1,074,918
non-oxidative hydroxyarylic acid decarboxylases subunit D	1,073,828	1,074,064	–	–	1,074,929	1,075,165
Hok/Gef family protein	1,074,403	1,074,555	–	–	1,075,504	1,075,656
DNA mismatch repair protein MutS	1,074,675	1,077,236	–	–	1,075,776	1,078,337
tyrosine-type recombinase/integrase	1,077,497	1,078,798	–	–	1,078,598	1,079,899
integrase domain-containing protein	1,078,941	1,079,816	–	–	1,080,042	1,080,917
hypothetical protein	1,079,843	1,080,157	–	–	1,080,944	1,081,258
hypothetical protein	1,080,664	1,080,864	–	–	1,081,765	1,081,965
hypothetical protein	1,080,875	1,081,612	–	–	1,081,976	1,082,713
hypothetical protein	1,081,913	1,083,040	–	–	1,083,014	1,084,141
hypothetical protein	1,083,070	1,083,537	–	–	1,084,171	1,084,638
primase-helicase family protein	1,083,723	1,085,399	–	–	1,084,824	1,086,500
helix-turn-helix transcriptional regulator	1,085,442	1,085,657	–	–	1,086,543	1,086,758
hypothetical protein	1,085,953	1,086,609	–	–	1,087,054	1,087,710
DUF4062 domain-containing protein	1,087,249	1,088,226	–	–	1,088,350	1,089,327
S8 family peptidase	1,088,368	1,090,857	–	–	1,089,469	1,091,958
AAA family ATPase	1,090,866	1,091,852	–	–	1,091,967	1,092,953
hypothetical protein	1,092,218	1,092,388	–	–	1,093,319	1,093,489
Fic/DOC family protein	1,092,385	1,093,002	–	–	1,093,486	1,094,103
heme ABC transporter ATP-binding protein	1,093,366	1,094,145	–	–	1,094,467	1,095,246
FecCD family ABC transporter permease	1,094,138	1,095,133	–	–	1,095,239	1,096,234
hemin ABC transporter substrate-binding protein	1,095,133	1,095,951	–	–	1,096,234	1,097,052
hemin-degrading factor	1,095,948	1,096,979	–	–	1,097,049	1,098,080
TonB-dependent receptor	1,097,050	1,099,413	–	–	1,098,151	1,100,514
SelT/SelW/SelH family protein	1,099,629	1,099,922	–	–	1,100,730	1,101,023
thiamine pyrophosphate-requiring protein	1,099,962	1,101,671	–	–	1,101,063	1,102,772
AEC family transporter	1,101,707	1,102,648	–	–	1,102,808	1,103,749
LysR family transcriptional regulator	1,102,833	1,103,720	–	–	1,103,934	1,104,821
nitrous oxide-stimulated promoter family protein	1,103,788	1,104,129	–	–	1,104,889	1,105,230
metal ABC transporter permease	1,104,196	1,105,050	–	–	1,105,297	1,106,151
iron/manganese ABC transporter permease subunit SitC	1,105,044	1,105,892	–	–	1,106,145	1,106,993
manganese/iron ABC transporter ATP-binding protein	1,105,889	1,106,710	–	–	1,106,990	1,107,811
metal ABC transporter substrate-binding protein	1,106,707	1,107,624	–	–	1,107,808	1,108,725
LacI family DNA-binding transcriptional regulator	1,107,803	1,108,765	–	–	1,108,904	1,109,866
ROK family protein	1,108,822	1,109,736	–	–	1,109,923	1,110,837
PTS lactose/cellobiose transporter subunit IIA	1,109,787	1,110,101	–	–	1,110,888	1,111,202
PTS cellobiose transporter subunit IIC	1,110,125	1,111,447	–	–	1,111,226	1,112,548
PTS sugar transporter subunit IIB	1,111,457	1,111,771	–	–	1,112,558	1,112,872
PTS transporter subunit EIIC	1,111,949	1,113,220	–	–	1,113,050	1,114,321
maltoporin	1,113,483	1,114,820	–	–	1,114,584	1,115,921
CBM35 domain-containing protein	1,114,833	1,117,025	–	–	1,115,934	1,118,126
glycoside hydrolase family 1 protein	1,117,077	1,118,477	–	–	1,118,178	1,119,578
formate hydrogenlyase transcriptional activator FlhA	1,118,531	1,120,603	–	–	1,119,632	1,121,704
hydrogenase expression/formation protein HypE	1,120,840	1,121,850	–	–	1,121,941	1,122,951
hydrogenase formation protein HypD	1,121,847	1,122,968	1,067,982*	1,068,413*	1,122,948	1,124,069
GNAT family N-acetyltransferase^3)^	1,396,328	1,398,331	–	–	1,397,429	1,399,432
tRNA(Met) cytidine acetyltransferase TmcA^3)^	–	–	1,341,773	1,343,776	–	–
NADH-quinone oxidoreductase subunit NuoA	1,569,954	1,570,400	1,515,399*	1,515,843*	1,571,055	1,571,501
undecaprenyl-phosphate glucose phosphotransferase wcaJ	1,788,502	1,789,899	1,733,945*	1,735,298*	1,789,603	1,791,000
**Plasmid**						
mucoid phenotype regulator RmpA	22,630	23,262	–	–	–	–
tetracycline resistance transcriptional repressor TetR(B)	–	–	22,808	23,431	22,808	23,431
tetracycline efflux MFS transporter Tet(B)	–	–	23,513	24,718	23,513	24,718

1) Not present, 2) Pseudogene, 3) Single amino acid substitution (238th T to A).

Compared with the WT, both recombinants harbored an additional chromosomal copy of ISKpn28 (Figure 4), located at the same position within the *nlpD* gene. Both recombinant derivatives retained the ISKpn28 loci present in parental K255. In K255Δ*rmpA*-2, the ISKpn28 copy was flanked by a 5-bp DR, “GGTGG” (Table 3, Site C). No adjacent flanking sequences were detected in the inserted segment, which consisted only of the ISKpn28 sequence. In K255Δ*rmpA*-1, the downstream region of *nlpD* was lost owing to the chromosomal deletion (Figure 4). In K255Δ*rmpA*-2, *nlpD* disruption resulted in two separate pseudogenes: one predicted to encode a “LysM peptidoglycan-binding domain-containing protein” and the other a “putative murein hydrolase activator NlpD” (Table 2). Junction-specific PCR using genomic DNA from parental K255 and the two recombinant derivatives confirmed the strain-specific junction patterns (Figure S2(A–C)). PCR analysis of 10 independently selected colonies from each recombinant derivative consistently reproduced the corresponding junction patterns, supporting clonal fixation of these genomic alterations (Figure S2(D–F)). PCR amplification and Sanger sequencing confirmed the examined ISKpn28-associated junctions in both recombinant strains (Figure S3).

**Figure 5.**
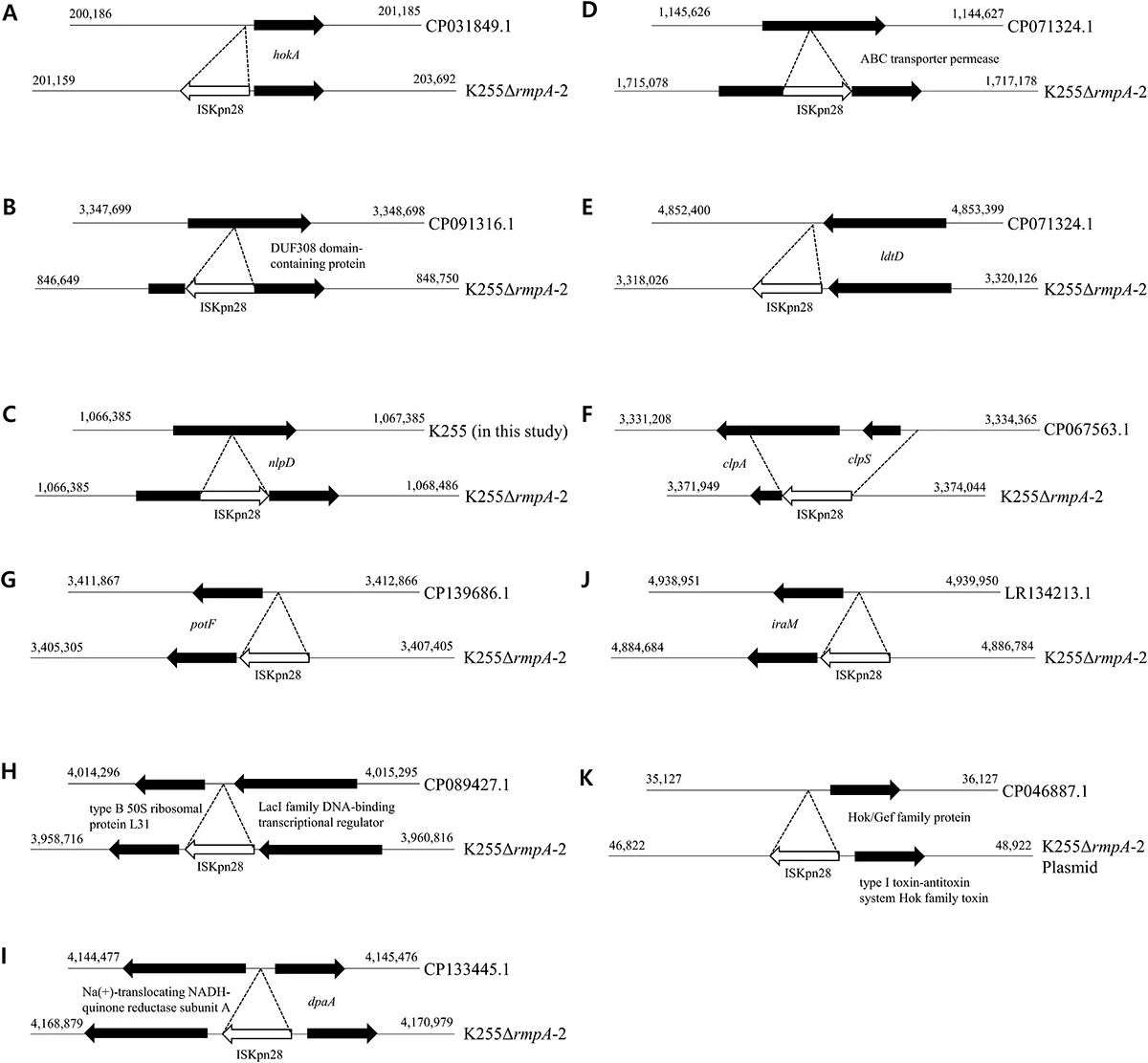
Genomic contexts of ISKpn28 loci in K255Δ*rmpA*-2. Schematic comparisons of the ISKpn28 loci in K255Δ*rmpA*-2 with the corresponding regions without ISKpn28 insertion in parental K255 or selected *K. pneumoniae* reference sequences. ISKpn28 loci in K255Δ*rmpA*-2 were located as follows: (A) upstream of *hokA*; (B) within a DUF308 domain-containing protein; (C) within *nlpD*; (D) within an ABC transporter permease gene; (E) downstream of *ldtD*; (F) within *clpA* and upstream of *clpS*; (G) upstream of *potF*; (H) upstream of the gene encoding type B 50S ribosomal protein L31; (I) upstream of the gene encoding Na(+)-translocating NADH-quinone reductase subunit A; (J) upstream of *iraM*; and (K) upstream of a Hok/Gef family protein-encoding gene on the plasmid. Black arrows represent annotated genes and their orientations, with disrupted or adjacent genes labeled. White arrows indicate the positions and orientations of ISKpn28. GenBank accession numbers of the reference sequences are indicated to the right. Numbers shown above and below the schematic indicate nucleotide coordinates in the corresponding sequences.

**Table 3.** ISKpn28 loci profiles in K255 recombinants.

Contig	Site^3)^	Coordinate (bp)	Orientation	Left flank	Direct Repeat	Right flank	Presumed pre-insertion site found in other strains (GenBank)
Chromosome	A	202,082 ~ 203207^1)^	-^4)^	GCCTCCAGTG	Not^6)^	AAGTGTTGAT	Upstream of *hokA* (CP031849.1)
		202,081 ~ 203206^2)^					
	B	847,140 ~ 848265^1)^	–	GTTAAAAAGA	TACCG	TAGAGCTACT	DUF308 domain-containing protein (CP091326.1)
		847,139 ~ 848264^2)^					
	C	1,066,871 ~ 1067996^1)^	+^5)^	ACCGCGGTGG	Not^6)^	ACCAAAAAAG	Middle of *nlpD* and *hypD* (K255, in this study)
		1,066,870 ~ 1067995^2)^		AATCGACCGC	GGTGG	TTGCTTCACA	*nlpD* (K255, in this study)
	D	1,659,905 ~ 1661030^1)^	+	GGCTGGCTGC	CGGCG	CTGATAGCCC	ABC transporter permease (CP071324.1)
		1,715,563 ~ 1716688^2)^					
	E	3,262,916 ~ 3264041^1)^	–	TGGGGTGAGG	CTGCC	TTGACGTTGG	Downstream of *ldtD* (CP071324.1)
		3,318,516 ~ 3319641^2)^					
	F	3,316,839 ~ 3317954^1)^	–	TAGATGATAG	Not^6)^	CCGGACGTGT	Upstream of *clpS*, middle of *clpA* (CP067563.1)
		3,372,439 ~ 3373554^2)^					
	G	3,350,195 ~ 3351320^1)^	–	TTTTTACCTG	GGCTA	TAGCCTCTTA	Upstream of *potF* (CP139686.1)
		3,405,795 ~ 3406920^2)^					
	H	3,903,746 ~ 3904871^1)^	–	TTAAATTGAG	CAGGG	TTGATCTTTG	Upstream of type B 50S ribosomal protein L31 (CP089427.1)
		3,959,206 ~ 3960331^2)^					
	I	4,113,909 ~ 4115034^1)^	–	TATCGCCCCT	TGCGC	TACAGTCGCC	Upstream of Na(+)-translocating NADH-quinone reductase subunit A (CP133445.1)
		4,169,369 ~ 4170494^2)^					
	J	4,829,714 ~ 4830839^1)^	–	AATTGTCAGG	GGTGC	TTGCGATTTA	Upstream of *iraM* (LR134213.1)
		4,885,174 ~ 4886299^2)^					
Plasmid	K	47,312 ~ 48437^1)^	–	ATAGCCTTTT	TCTCC	TTGCCTTACG	Upstream of Hok/Gef family protein (CP046887.1)
		47,312 ~ 48437^2)^					

1) K255Δ*rmpA*-1, 2) K255Δ*rmpA*-2, 3) Site position corresponds to Figure 5 and 4) Negative strand, 5) Positive strand, 6) Not detected.

At another locus, a region annotated as a GNAT family N-acetyltransferase in the WT and K255Δ*rmpA*-2 was annotated as a tRNA(Met) cytidine acetyltransferase TmcA in K255Δ*rmpA*-1 (Table 2). In both recombinants, the plasmid-borne *rmpA* gene was replaced by a tetracycline resistance cassette.

Additionally, K255Δ*rmpA*-2 exhibited a plasmid inversion relative to the WT plasmid, flanked by ISKpn74 sequences, without altering gene content. The parental and inverted plasmid configurations were further examined by configuration-specific junction PCR in all three strains (Figure S4(A, B)). Junctions corresponding to the parental configuration were detected in K255 and K255Δ*rmpA*-1, whereas inversion-specific junctions were detected only in K255Δ*rmpA*-2. The parental configuration was consistently detected in all 10 K255Δ*rmpA*-1 colonies examined, whereas the inverted configuration was detected in all 10 K255Δ*rmpA*-2 colonies examined (Figure S4C, D). Sanger sequencing verified the identities of the corresponding ISKpn74-containing PCR amplicons. Sanger sequencing files and inversion-junction sequence alignments are available in the Figshare repository [18].

Long-read mapping showed alignment discontinuities near the predicted inversion boundaries in K255Δ*rmpA*-2, whereas these patterns were not observed in K255 or K255Δ*rmpA*-1 (Figure S5(A)). SA-tagged alignments were detected at higher counts in the four regions flanking the predicted inversion boundaries in K255Δ*rmpA*-2 (Figure S5(B)).

In K255Δ*rmpA*-2, comparison of the assemblies generated before and after removal of the anomalous read showed minor differences in assembly metrics, whereas the identified structural alterations, including the additional ISKpn28 copy and ISKpn74-flanked plasmid inversion, remained unchanged (Table S4).

### Genomic contexts of ISKpn28 loci

As illustrated in Figure 5(A–K), a total of 11 ISKpn28 loci identified in the K255Δ*rmpA*-2 strain were observed in diverse genomic contexts: three loci were located within annotated coding regions, seven were located in intergenic regions, and the remaining locus was located at a *clpA-clpS* deletion junction. At this locus, which lacked flanking DRs (Figure 5(F)), the left flank sequence corresponded to the downstream region of *clpA*, whereas the right flank sequence corresponded to the upstream region of *clpS*. The corresponding region in K255Δ*rmpA*-2 was 2,162 bp shorter than that in *K. pneumoniae* strain ARLG-10778 (GenBank accession no. CP067563.1), with the ISKpn28 element located at the deletion junction. Similarly, in K255Δ*rmpA*-1, the left flank and corresponding DR originated from *nlpD*, whereas the right flank and DR were located within *hypD*, with no intact DR pair identified (Table 3, Site C).

## Discussion

This study unexpectedly identified genomic evidence of ISKpn28 transposition and secondary genomic alterations in λ-Red-derived recombinants of the hvKp strain K255. Two distinct recombinants displayed divergent phenotypes and in vitro virulence-associated traits despite carrying the same PCR- and Sanger sequencing-confirmed replacement of *rmpA* with a tetracycline-resistance cassette.

Comparison of mucoviscosity and uronic acid content under tetracycline-free and tetracycline-containing conditions allowed evaluation of the influence of tetracycline exposure. Notably, the difference in mucoviscosity was observed only under tetracycline-containing conditions, whereas the higher uronic acid content of K255Δ*rmpA*-2 persisted under both conditions. However, because both recombinant strains retained the tetracycline-resistance cassette, polar or transcriptional effects of the cassette could not be excluded.

Moreover, the two recombinant strains harbored multiple secondary genomic alterations. The observed phenotypic differences could not be attributed specifically to *rmpA* replacement, an additional copy of ISKpn28 in *nlpD*, or any single genomic alteration. Further studies using clean isogenic mutants and complemented strains will be required to determine the contribution of individual genetic alterations and to assess the potential phenotypic and pathogenic consequences of ISKpn28 transposition.

ISKpn28, a member of the IS481 family [21], has previously been identified in association with *mgrB* disruption and colistin resistance in *K. pneumoniae* [28], as well as with prophage diversity [29]. However, these previous studies did not specifically address the possibility of ISKpn28 transposition. In contrast, this study provides parent–derivative comparative genomic evidence of ISKpn28 transposition, based on the additional *nlpD*-associated ISKpn28 copy identified in both recombinant derivatives relative to parental K255. In K255Δ*rmpA*-2, the additional copy consisted solely of ISKpn28, was flanked by a 5-bp DR, and coexisted with the ISKpn28 loci present in parental K255. This configuration is consistent with a copy-and-paste transposition model proposed for the IS481 family [21,30].

A key strength of this study was the direct comparison of the parental strain and its recombinant derivatives using WGS incorporating both short- and long-read data, enabling genome-wide assessment beyond locus-specific validation. However, only two recombinant strains derived from a single parental strain and one mutant-construction experiment were examined. Therefore, the recurrence, frequency, temporal sequence, insertion-site preference, molecular mechanisms, and broader biological significance of these ISKpn28-associated events could not be determined. It also remains unclear whether the observed chromosomal deletion and plasmid inversion arose spontaneously or were associated with the λ-Red-mediated mutant-construction process. In addition, the phenotypic assays used in this study represent in vitro virulence-associated phenotypes and do not establish differences in virulence or pathogenic potential.

Nevertheless, these findings indicate that routine PCR- and Sanger sequencing-based confirmation of the intended λ-Red recombination may be insufficient to exclude unintended genomic alterations elsewhere in the genome. Therefore, WGS incorporating long-read data may be considered when validating λ-Red recombinants derived from hvKp strains harboring ISKpn28, before phenotypic differences are attributed to the targeted genetic modification.

## Supplementary Material

Figure S4_1.TIF

Figure S4_3.TIF

Figure S3_1.TIF

Supplementary_Figure_legends.docx

Figure S1.tif

Figure S2_3.TIF

Figure S5_2.TIF

Supplementary Table 3_Primers used in this study and PCR conditions.docx

Figure S4_2.TIF

Supplementary Table 4_Comparison of key assembly and annotation features before and after removal of the anomalous read in K255deltarmpA_2.docx

KVIR_OpenScienceDisclosureForm.docx

Supplementary Table 5_Plasmid inversion regions and SAMtools commands used to count SA_tagged alignments.docx

Figure S3_4.TIF

Figure S2_1.TIF

Figure S3_3.TIF

Figure S5_1.TIF

Supplementary Table 1_Bacterial strains used in this study.docx

Supplementary Table 2_Plasmids used in this study.docx

Supplementary Table 6_Hypothetical protein difference.docx

Figure S2_2.TIF

Figure S2_4.TIF

Figure S3_2.TIF

## Data Availability

The sequencing datasets generated in this study have been deposited in the NCBI BioProject database (https://www.ncbi.nlm.nih.gov/bioproject/PRJNA1464084/). All other raw data and supplementary information associated with this manuscript, including raw numerical data underlying the figures, statistical analyses, raw Sanger sequencing data, junction sequence alignments, Kleborate results, original colony morphology images, PGAP annotation results, Supplementary Tables S1–S6, Supplementary Figures S1–S5, and supplementary figure legends, are publicly available in the Figshare repository [18] at https://doi.org/10.6084/m9.figshare.32293899 under a CC BY 4.0 license.

## Open scholarship statement

This article has earned the Center for Open Science badge for Open Data. The data are openly accessible at https://doi.org/10.6084/m9.figshare.32293899
