# Supplementary material for: ISKpn28 transposition and genomic alterations in λ-Red recombinants of hypervirulent Klebsiella pneumoniae: Whole-genome sequencing and phenotypic analysis
Source: Virulence. 2026 Nov 1;17(1):2732382. doi: 10.1080/21505594.2026.2732382 (PMC13733257; doi:10.1080/21505594.2026.2732382)
Supplement: Supplementary file 1 [file KVIR_A_2732382_SM4643.docx]

| Chromosome | | | | | | |
| --- | --- | --- | --- | --- | --- | --- |
| Product | K255 | | K255Δ*rmpA*-1 | | K255Δ*rmpA*-2 | |
|  | Start | Stop | Start | Stop | Start | Stop |
| Hypothetical protein | 2413994 | 2414152 | 2359393* | 2359551* | 2414993* | 2415151* |
| Plasmid | | | | | | |
| Hypothetical protein | 132545 | 132691 | 134184* | 134330* | 75858 | 76004 |
| Hypothetical protein | 168267 | 168449 | 169914* | 170096* | 169906 | 170088 |

Supplementary Table S6. Hypothetical protein annotation difference

* : Not annotated
