# Supplementary material for: ISKpn28 transposition and genomic alterations in λ-Red recombinants of hypervirulent Klebsiella pneumoniae: Whole-genome sequencing and phenotypic analysis
Source: Virulence. 2026 Nov 1;17(1):2732382. doi: 10.1080/21505594.2026.2732382 (PMC13733257; doi:10.1080/21505594.2026.2732382)
Supplement: Supplementary file 2 [file KVIR_A_2732382_SM4644.docx]

| **Plasmid** | **Antibiotic Resistance** | **Purpose** | **Source** |
| --- | --- | --- | --- |
| pKD46 | Spectinomycin | λ-red recombination system | (1) |

Supplementary Table S2. Plasmids used in this study

1.	Datsenko KA, Wanner BL. One-step inactivation of chromosomal genes in *Escherichia coli* K-12 using PCR products. Proceedings of the National Academy of Sciences. 2000;97(12):6640–5.
