# Supplementary material for: ISKpn28 transposition and genomic alterations in λ-Red recombinants of hypervirulent Klebsiella pneumoniae: Whole-genome sequencing and phenotypic analysis
Source: Virulence. 2026 Nov 1;17(1):2732382. doi: 10.1080/21505594.2026.2732382 (PMC13733257; doi:10.1080/21505594.2026.2732382)
Supplement: Supplementary file 3 [file KVIR_A_2732382_SM4645.docx]

| **Strain** | **Purpose** | **Source** |
| --- | --- | --- |
| K255 | Wild type strain used in this study | Keimyung University Dongsan Hospital |
| K255Δ*rmpA*-1 | *rmpA* gene was replaced with a tetracycline resistance cassette in this study | K255 |
| K255Δ*rmpA*-2 |  | K255 |
| Salmonella enterica sv Typhimurium strain LT2 | Tetracycline resistance gene cassette template | ATCC 700720 |

Supplementary Table S1. Bacterial strains used in this study
