# Supplementary material for: ISKpn28 transposition and genomic alterations in λ-Red recombinants of hypervirulent Klebsiella pneumoniae: Whole-genome sequencing and phenotypic analysis
Source: Virulence. 2026 Nov 1;17(1):2732382. doi: 10.1080/21505594.2026.2732382 (PMC13733257; doi:10.1080/21505594.2026.2732382)
Supplement: Supplementary file 4 [file KVIR_A_2732382_SM4650.docx]

**Supplementary Table S5. Plasmid inversion regions and SAMtools commands used to count SA-tagged alignments**

| Schematic Diagram | | |
| --- | --- | --- |
| 1 2 3 4  ISKpn74  Inverted region  ISKpn74 | | |
| Region | Strain | Command |
| 1 | K255 | samtools view K255.bam 2:67745-72745 \| grep -c 'SA:Z:' |
|  | K255Δ*rmpA*-1 | samtools view 1.bam 2:67745-72745 \| grep -c 'SA:Z:' |
|  | K255Δ*rmpA*-2 | samtools view 2.bam 2:67745-72745 \| grep -c 'SA:Z:' |
| 2 | K255 | samtools view K255.bam 2:73802-78802 \| grep -c 'SA:Z:' |
|  | K255Δ*rmpA*-1 | samtools view 1.bam 2:73802-78802 \| grep -c 'SA:Z:' |
|  | K255Δ*rmpA*-2 | samtools view 2.bam 2:73802-78802 \| grep -c 'SA:Z:' |
| 3 | K255 | samtools view K255.bam 2:128108-133108 \| grep -c 'SA:Z:' |
|  | K255Δ*rmpA*-1 | samtools view 1.bam 2:128108-133108 \| grep -c 'SA:Z:' |
|  | K255Δ*rmpA*-2 | samtools view 2.bam 2:128108-133108 \| grep -c 'SA:Z:' |
| 4 | K255 | samtools view K255.bam 2:134165-139165 \| grep -c 'SA:Z:' |
|  | K255Δ*rmpA*-1 | samtools view 1.bam 2:134165-139165 \| grep -c 'SA:Z:' |
|  | K255Δ*rmpA*-2 | samtools view 2.bam 2:134165-139165 \| grep -c 'SA:Z:' |

- In the BAM file names, “1” and “2” indicate K255Δ*rmpA*-1 and K255Δ*rmpA*-2, respectively.
- Regions 1–4 correspond to the regions indicated in the schematic diagram.
