# Supplementary material for: ISKpn28 transposition and genomic alterations in λ-Red recombinants of hypervirulent Klebsiella pneumoniae: Whole-genome sequencing and phenotypic analysis
Source: Virulence. 2026 Nov 1;17(1):2732382. doi: 10.1080/21505594.2026.2732382 (PMC13733257; doi:10.1080/21505594.2026.2732382)
Supplement: Supplementary file 6 [file KVIR_A_2732382_SM4653.docx]

Supplementary Table S4. Comparison of key assembly and annotation features before and after removal of the anomalous read in K255Δ*rmpA*-2

**Removed anomalous read sequence in short-read raw data of K255Δ*rmpA*-2**

Read header: @LH00684:5:22NFYWLT3:1:2104:22793:18897 1:N:0:GTACGTAC+GAGTCGCT

AAACTATATCTATCTCGATCACTAAGCCGGCGGGGGGGGGGGGGGGGGAAAAAAACACCCCCCCACCCAGCCTCCCCCCCCCCCCCCCCCCCCCCCCCCCCCCCCCCCCCCCCCCCCCCCCCCAAACCCCCCCCCCCCCCCCCCCCCCCCC

*: The poly-C tract in the raw read appears as a poly-G tract in the chromosome assembly because of its reverse-complement orientation.

| **Comparison of key assembly and annotation features before and after anomalous read removal** | | | |
| --- | --- | --- | --- |
| **Feature** | **Before anomalous-read removal** | **After anomalous-read removal** | **Interpretation** |
| Chromosome length | 5,253,433 bp | 5,253,470 bp | Comparable |
| Plasmid length | 236,588 bp | 236,593 bp | Comparable |
| Total genes | 5,365 | 5,365 | Unchanged |
| Pseudogenes | 146 | 143 | Decreased |
| *nlpD*::ISKpn28 region | Detected | Detected | Retained |
| ISKpn28 loci relevant to this study | Retained (11 loci) | Retained (11 loci) | Not affected |
| ISKpn74-associated plasmid inversion | Detected | Detected | Retained |
| Anomalous polyG* sequence in assembly | Present  (in chromosome) | Removed | Targeted filtering |
