# Supplementary material for: ISKpn28 transposition and genomic alterations in λ-Red recombinants of hypervirulent Klebsiella pneumoniae: Whole-genome sequencing and phenotypic analysis
Source: Virulence. 2026 Nov 1;17(1):2732382. doi: 10.1080/21505594.2026.2732382 (PMC13733257; doi:10.1080/21505594.2026.2732382)
Supplement: Supplementary file 7 [file KVIR_A_2732382_SM4655.docx]

Supplementary Table S3. Primers used in this study and PCR conditions

| **Primer Name** | **Sequence (5’ to 3’)** | **Purpose** | **Source** |
| --- | --- | --- | --- |
| rmpA-F Kleb plasmid 35357 | GGATCAAAGTTACTGTTTCTATGGAAAAATATATTTACTTTATATGTGCAGGCTGGAGCTGCTTC | Amplification of tetracycline resistance cassette for *rmpA* replacement | This study |
| rmpA-R Kleb plasmid 34703 | ACCTAAATACTTGGCATGAGCCATCTTTCATCAACCATTTCTTTACATATGAATATCCTCCTTAG |  | This study |
| rmpA_Kleb_outer_primer_F | CGCGAGCGGAAGATTCTTTT | PCR and Sanger sequencing validation of recombination | This study |
| rmpA_Kleb_outer_primer_R | TGCAAACACGCAAAGACAAA |  | This study |
| Cassette-R | CTTCGAAGCAGCTCCAGCCTGCAC |  | (1) |
| nlpD_F | TTACAACCGCAAATATGGC | PCR and Sanger sequencing validation of junction sequences | This study |
| ISKpn28_up_R | TTGCTTCTTGGAATTAGTGC |  | This study |
| ISKpn28_down_F | CCTCGATTAAACACATCTGG |  | This study |
| hypD_R | GCGTTACGTAGATGAATACC |  | This study |
| nlpD_R | GTAGGCACTCAGGTAATCAT |  | This study |
| left_ISKpn74_F | TCAAGCATTTCCTAGACTGC | PCR and Sanger sequencing validation of plasmid inversion | This study |
| inversion_seq | AGGGGACTTTCTGTTTCTTC |  | This study |
| right_ISKpn74_R | TGCTTCACTCAGTAGATTGG |  | This study |

1.	Mukhtar M, Ghafoor A, McClelland M, Akhtar F, Rasheed MA. Construction, molecular characterization, and safety assessment of *purB* mutant of *Salmonella Gallinarum*. Front Microbiol. 2024;15:1467230.

**PCR reaction conditions**

| Figure | | **Figure 1A** | **Figure 1B** |
| --- | --- | --- | --- |
| PCR mixture (per reaction) | | | |
| Primer combination | | F: rmpA_Kleb_outer_primer_R  R: rmpA_Kleb_outer_primer_F | F: rmpA_Kleb_outer_primer_R  R: Cassette-R |
| Template (µL) | | 2 | |
| Primer Mix (µL) | | 6 | |
| MgCl_2_ (µL) | | 5 | |
| dNTP (µL) | | 1 | |
| DNA polymerase (µL) | | 0.25 | |
| Buffer (µL) | | 10 (Colorless) | |
| DW | | Up to 50 µL | |
| Final concentration of each primer set | | Each 0.3 µM | |
| Thermal cycling conditions | | | |
| 95 ℃ | | 2 min | 2 min |
| 95 ℃ | X 30 | 30 sec | 30 sec |
| 56 ℃ |  | 15 sec | 15 sec |
| 72 ℃ |  | 2 min 35 sec | 15 sec |
| 72 ℃ | | 5 min | 5 min |
| 4 ℃ | | ∞ | ∞ |

| Figure | | **Figure S2A** | **Figure S2B** | **Figure S2C** |
| --- | --- | --- | --- | --- |
| PCR mixture (per reaction) | | | | |
| Primer combination | | F: nlpD_F  R: ISKpn28_up_R | F: ISKpn28_down_F  R: nlpD_R | F: ISKpn28_down_F  R: hypD_R |
| Template (µL) | | 2 | | |
| Primer Mix (µL) | | 10 | | |
| MgCl_2_ (µL) | | 5 | | |
| dNTP (µL) | | 1 | | |
| DNA polymerase (µL) | | 0.25 | | |
| Buffer (µL) | | 10 (Colorless) | | |
| DW | | Up to 50 µL | | |
| Final concentration of each primer set | | Each 0.5 µM | | |
| Thermal cycling conditions | | | | |
| 95 ℃ | | 2 min | | |
| 95 ℃ | X 30 | 30 sec | | |
| 51 ℃ |  | 30 sec | | |
| 72 ℃ |  | 50 sec | | |
| 72 ℃ | | 5 min | | |
| 4 ℃ | | ∞ | | |

| Figure | | **Figure S2D** | **Figure S2E** | **Figure S2F** |
| --- | --- | --- | --- | --- |
| PCR mixture (per reaction) | | | | |
| Primer combination | | F: nlpD_F  R: ISKpn28_up_R | F: ISKpn28_down_F  R: nlpD_R | F: ISKpn28_down_F  R: hypD_R |
| Template (µL) | | 0.5 | | |
| Primer Mix (µL) | | 1 | | |
| MgCl_2_ (µL) | | 0.5 | | |
| dNTP (µL) | | 0.1 | | |
| DNA polymerase (µL) | | 0.025 | | |
| Buffer (µL) | | 1 (Colored) | | |
| DW | | Up to 5 µL | | |
| Final concentration of each primer set | | Each 0.5 µM | | |
| Thermal cycling conditions | | | | |
| 95 ℃ | | 2 min | | |
| 95 ℃ | X 30 | 30 sec | | |
| 51 ℃ |  | 30 sec | | |
| 72 ℃ |  | 50 sec | | |
| 72 ℃ | | 5 min | | |
| 4 ℃ | | ∞ | | |

| Figure | | **Figure S4A** | **Figure S4B** |
| --- | --- | --- | --- |
| PCR mixture (per reaction) | | | |
| Primer combination | | F: left_ISKpn74_F  R: inversion_seq | F: inversion_seq  R: right_ISKpn74_R |
| Template (µL) | | 2 | |
| Primer Mix (µL) | | 6 | |
| MgCl_2_ (µL) | | 5 | |
| dNTP (µL) | | 1 | |
| DNA polymerase (µL) | | 0.25 | |
| Buffer (µL) | | 10 (Colorless) | |
| DW | | Up to 50 µL | |
| Final concentration of each primer set | | Each 0.3 µM | |
| Thermal cycling conditions | | | |
| 95 ℃ | | 2 min | |
| 95 ℃ | X 30 | 30 sec | |
| 53 ℃ |  | 15 sec | |
| 72 ℃ |  | 2 min | |
| 72 ℃ | | 5 min | |
| 4 ℃ | | ∞ | |

| Figure | | **Figure S4C** | **Figure S4D** |
| --- | --- | --- | --- |
| PCR mixture (per reaction) | | | |
| Primer combination | | F: left_ISKpn74_F  R: inversion_seq | F: inversion_seq  R: right_ISKpn74_R |
| Template (µL) | | 0.5 | |
| Primer Mix (µL) | | 0.6 | |
| MgCl_2_ (µL) | | 0.5 | |
| dNTP (µL) | | 0.1 | |
| DNA polymerase (µL) | | 0.025 | |
| Buffer (µL) | | 1 (Colored) | |
| DW | | Up to 5 µL | |
| Final concentration of each primer set | | Each 0.3 µM | |
| Thermal cycling conditions | | | |
| 95 ℃ | | 2 min | |
| 95 ℃ | X 30 | 30 sec | |
| 53 ℃ |  | 15 sec | |
| 72 ℃ |  | 2 min | |
| 72 ℃ | | 5 min | |
| 4 ℃ | | ∞ | |
