# Supplementary material for: ISKpn28 transposition and genomic alterations in λ-Red recombinants of hypervirulent Klebsiella pneumoniae: Whole-genome sequencing and phenotypic analysis
Source: Virulence. 2026 Nov 1;17(1):2732382. doi: 10.1080/21505594.2026.2732382 (PMC13733257; doi:10.1080/21505594.2026.2732382)
Supplement: Supplementary file 8 [file KVIR_A_2732382_SM4659.docx]

**Figure S1. String test results of the recombinant strains under tetracycline-free and tetracycline-containing conditions.**

String lengths of K255Δ*rmpA*-1 and K255Δ*rmpA*-2 grown on LB agar without tetracycline (A) or with tetracycline (15 μg/mL) (B). Each point represents an individual colony.

**Figure S2. Junction-specific PCR validation and clonal fixation of the additional *nlpD*-associated ISKpn28 copy and the 55,652-bp deletion.**

(A–C) Junction-specific PCR analysis of parental K255 and the two recombinant derivatives. (A) The upstream *nlpD*–ISKpn28 junction was detected in K255Δ*rmpA*-1 and K255Δ*rmpA*-2. (B) The downstream *nlpD*–ISKpn28 junction was detected only in K255Δ*rmpA*-2. (C) The ISKpn28–*hypD* deletion junction was detected only in K255Δ*rmpA*-1. Schematics show the names and positions of the primers, and dashed boxes indicate the amplified junctions. (D–F) PCR of ten separately selected colonies from each recombinant derivative. (D) The upstream *nlpD*–ISKpn28 junction was detected in all colonies of both derivatives. (E) The downstream *nlpD*–ISKpn28 junction was detected in all K255Δ*rmpA*-2 colonies but not in K255Δ*rmpA*-1 colonies. (F) The ISKpn28–*hypD* deletion junction was detected in all K255Δ*rmpA*-1 colonies but not in K255Δ*rmpA*-2 colonies. M, DNA size marker; WT, parental K255; Δ1, K255Δ*rmpA*-1; Δ2, K255Δ*rmpA*-2.

**Figure S3. Sanger sequencing validation of the ISKpn28-associated junctions in the recombinant strains.**

Consensus sequence alignments and bidirectional Sanger sequencing chromatograms of (A) the upstream *nlpD*–ISKpn28 junction in K255Δ*rmpA*-1, (B) the ISKpn28–*hypD* deletion junction in K255Δ*rmpA*-1, (C) the upstream *nlpD*–ISKpn28 junction in K255Δ*rmpA*-2, and (D) the downstream *nlpD*–ISKpn28 junction in K255Δ*rmpA*-2. The yellow highlighted regions indicate the predicted junction-spanning sequences.

**Figure S4. Configuration-specific PCR validation and clonal fixation of the ISKpn74-flanked plasmid inversion.**

(A, B) Configuration-specific PCR analysis of parental K255 and the two recombinant derivatives. (A) The parental-configuration-specific amplicon was detected in K255 and K255Δ*rmpA*-1 but not in K255Δ*rmpA*-2. (B) The inversion-specific amplicon was detected only in K255Δ*rmpA*-2. Schematics depict the orientations of the ISKpn74-flanked plasmid region, with the primer names and positions shown for each configuration. (C, D) PCR of ten separately selected colonies from each recombinant derivative. (C) The parental-configuration-specific product was detected in all K255Δ*rmpA*-1 colonies but not in K255Δ*rmpA*-2 colonies. (D) The inversion-specific product was detected in all K255Δ*rmpA*-2 colonies but not in K255Δ*rmpA*-1 colonies. M, DNA size marker; WT, parental K255; Δ1, K255Δ*rmpA*-1; Δ2, K255Δ*rmpA*-2.

**Figure S5. Long-read mapping support for the ISKpn74-flanked plasmid inversion in K255Δ*rmpA*-2.**

(A) Visualization of long-read alignments from parental K255, K255Δ*rmpA*-1, and K255Δ*rmpA*-2 mapped to the 234-kb K255 reference plasmid. K255Δ*rmpA*-2 showed distinct alignment discontinuities at approximately 74 and 136 kb, near the predicted inversion boundaries. This alignment pattern was consistent with the predicted inverted plasmid configuration. (B) Schematic representation of the plasmid region flanked by two ISKpn74 copies. Numbers indicate the counts of supplementary alignments carrying SA tags in four 5-kb regions surrounding the predicted inversion boundaries. SA-tagged alignments were detected at higher counts in K255Δ*rmpA*-2 than in parental K255 and K255Δ*rmpA*-1, providing additional read-level support for the inverted configuration. Blue boxes indicate ISKpn74 copies, magenta arrows indicate the orientation of the intervening plasmid region, dashed lines delimit the analysed 5-kb regions, and double-headed arrows indicate the spans of these regions.
